# Aging Induced p53/p21 in Genioglossus Muscle Stem Cells and Enhanced Upper Airway Injury

**DOI:** 10.1155/2020/8412598

**Published:** 2020-03-04

**Authors:** Lu-Ying Zhu, Li-Ming Yu, Wei-Hua Zhang, Jia-Jia Deng, Shang-Feng Liu, Wei Huang, Meng-Han Zhang, Yan-Qin Lu, Xin-Xin Han, Yue-Hua Liu

**Affiliations:** ^1^Xiangya School of Stomatology, Xiangya Stomatological Hospital, Central South University, Changsha 410078, China; ^2^Oral Biomedical and Engineering Laboratory, Shanghai Stomatological Hospital, Fudan University, Shanghai 200001, China; ^3^Department of Orthodontics, Shanghai Stomatological Hospital, Fudan University, Shanghai 200001, China

## Abstract

Aging of population brings related social problems, such as muscle attenuation and regeneration barriers with increased aging. Muscle repair and regeneration depend on muscle stem cells (MuSCs). Obstructive sleep apnea (OSA) rises in the aging population. OSA leads to hypoxia and upper airway muscle injury. However, little is known about the effect of increasing age and hypoxia to the upper airway muscle. The genioglossus (GG) is the major dilator muscle to keep the upper airway open. Here, we reported that muscle fiber and MuSC function declined with aging in GG. Increasing age also decreased the migration and proliferation of GG MuSCs. p53 and p21 were high expressions both in muscle tissue and in GG MuSCs. We further found that hypoxia inhibited GG MuSC proliferation and decreased myogenic differentiation. Then, hypoxia enhanced the inhibition effect of aging to proliferation and differentiation. Finally, we investigated that hypoxia and aging interact to form a vicious circle with upregulation of p53 and p21. This vicious hypoxia plus aging damage accelerated upper airway muscle injury. Aging and hypoxia are the major damage elements in OSA patients, and we propose that the damage mechanism of hypoxia and aging in GG MuSCs will help to improve upper airway muscle regeneration.

## 1. Introduction

The root source of obstructive sleep apnea (OSA) is repeated hypoxia during sleep [1, 2], and OSA has a higher prevalence at advanced age [3, 4]. Genioglossus (GG), a major upper airway dilator, is key to OSA pathophysiology. Compared with other skeletal muscles, genioglossus has high specific gravity of oxidized muscle fiber and is sensitive to oxygen [5]. The upper airway muscle collapses more easily with aging [6], and there is an age-related change in the fiber-type distribution of the upper airway muscle [7]. However, the effect of increasing age to GG function and the related mechanism remains to be elucidated.

Muscle stem cells (MuSCs) are responsible for muscle growth and injury repair throughout the life [8]. After stimuli, MuSCs can differentiate into myocytes and then fuse with each other to repair damaged muscle [9, 10]. Muscle is a homeostatic tissue and can tolerate daily wear-and-tear by repair and regeneration [11]. With increasing age, the important reason of progressive weaken and regenerative dysfunction is the functional decline muscle MuSCs [12]. In aging cells, there are also inactivated antioxidative pathways, increased reactive oxygen species, and apoptosis [13]. GG repair and regeneration are very important to OSA patients. However, the influence of aging to GG MuSCs is still unknown.

p53 is a famous tumor suppressor and mutated in a large proportion of cancers [14]. Meanwhile, p53 is a transcription factor involved in many cell processes, such as cell-cycle control, DNA repair, apoptosis, and cellular stress responses [15]. p53 also is a downstream member of aging and hypoxia signaling pathway [16, 17]. p53 increases its expansion and encourages in aging skeletal muscle. An apoptotic environment is encouraged by p53 in muscle tissue [18]. Then, p53 is sensitive to hypoxia and may suppress muscle cell proliferation by interacting with p21 and hypoxia-inducible factor-1*α* (HIF-1*α*) [19]. p21 plays an important role in muscle differentiation after injury [20]. Limited reports show that hypoxia promotes autophagy and modulates mitochondrial function of the GG MuSCs [1, 2]. However, the mechanism of aging GG MuSCs under hypoxia and whether p53 and p21 are involved in this process are still unclear.

Repeated airway collapse and obstruction caused hypoxia in OSA patients. This hypoxia aggravates upper airway muscle damage. Muscle damage further increases obstruction and forms a vicious cycle. Some studies have reported that the prevalence of OSA increases with aging [3, 4]. Our previous work has showed that hypoxia inhibits the myogenic differentiation of GG MuSCs and causes muscle disturbances [21–23]. However, the mechanism of aging and hypoxia damage to GG MuSCs has few reported. In this study, we hypothesized that aging and hypoxia might injure the GG MuSCs by upregulating p53 and p21.

## 2. Materials and Methods

### 2.1. Animals and Ethical Issues

C57BL/6 mice (male, 1 to 12 months old) were obtained from Shanghai Bikai Biotechnology. Mice were kept under natural aging conditions in the animal house facility, with a 12:12 h light and dark cycle. All animals were anesthetized and euthanized. The mouse GG were removed, then frozen in liquid nitrogen and stored at −80°C rapidly for subsequent quantitative polymerase chain reaction (qPCR) measurements. This research complied with the Animal Ethics Committee of Shanghai Stomatology Hospital, Fudan University.

### 2.2. Cell Cultures and Proliferation Assays

Under sterile conditions, the GG were excised. Firstly, the tissues were cut into 1 mm^3^ size. Next, the muscle slurry was digested with 0.1% type I collagenase (Gibco, USA) and 0.05% trypsin-EDTA (Gibco, Canada) at 37°C each for 30 min. Then, the digestion was stopped by the addition of Dulbecco's Modified Eagle Medium (DMEM, Gibco, UK) supplemented with 10% fetal bovine serum (FBS, Gibco, New Zealand). Finally, cells were plated on the culture dishes, and twice repeated differential attachment treatment was used to remove fibroblasts. In the next experiments, to avoid fibroblasts taking over the other cell populations and becoming the predominant cell type in the culture, we only used MuSCs from passage 1. Once the cells reached 90% confluence, they were differentiated by incubation 2% horse serum (Hyclon, USA) in DMEM. CoCl_2_ was dissolved to 200 *μ*M for actual use in DMEM.

The proliferation of GG MuSCs was assessed using cell counting kit-8 (CCK-8, Dojindo, Japan) assays. Briefly, cells were seeded in 96-well plates at a density of 5 × 10^3^ cells per well. After 6 days culture, cells were treated with 10% CCK8 in DMEM for 2 h. Optical density (OD) of each well was measured at 430 nm on a microplate reader at 37°C.

### 2.3. Wound Healing Assay and Transwell Cell Migration Assay

For wound healing assay, GG MuSCs from four age groups were seeded in 6 well plates. After nearly100% confluence, a single wound was created with a sterile 200 *μ*l plastic pipette tip in the center of the well, then washed with PBS twice to remove the cellular debris and cultured by 1% FBS in DMEM for 24 h. The wound was captured at 0 and 24 h. The size of the wound healing was measured using Image J 1.5 software.

For migration assay, GG MuSCs (1 × 10^5^) were seeded in the transwell inserts (Costar, China, pore size: 8 mm). The assays and counting of migrating cells were performed as described previously [24]. After incubation at 37°C for 24 h, GG MuSCs remaining on the upper chamber membrane were removed with cotton swabs. The migrated cells were fixed in ice-cold 4% PFA for 10 min and stained with a 1% crystal violet solution for 10 min. Images were captured five field at 100× magnification.

### 2.4. Electromyography of the GG Muscle (EMG_GG_)

EMG_GG_ was acquired and analyzed as previously described [25]. In brief, mice were anesthetized with 1% pentobarbital, then we turned over the digastric muscle and exposed the genioglossus muscle. Next, two Teflon-insulated wire loop electrodes were used to record EMG_GG_. The EMG_GG_ signal was amplified, band-pass filtered from 1 to 1000 Hz (ADInstrument Australia), and digitized at a sampling rate of 1000 Hz (LabChart 8). The EMG_GG_ was rectified, and a 1 s time constant was applied to compute the moving average (LabChart 8).

### 2.5. Hematoxylin and Eosin and Masson Trichrome Staining

The GG were collected from four age groups male C57BL/6 mice and were fixed in ice-cold 4% PFA. Then, GG were embedded in paraffin and cut into 4 *μ*m thick sections by a paraffin slicer. Sections were mounted on glass slides, then were stained with hematoxylin and eosin (H&E, Solarbio, Beijing, China) staining for observing the muscle fiber morphology, and Masson trichrome staining (Servicebio, Wuhan, China) was performed to analyze collagen content in muscle fibers.

### 2.6. Immunohistochemistry (IHC) and Immunofluorescence (IF) Assay of Tissues

For IHC assay, the 4 *μ*m sections embedded in paraffin were deparaffinized and rehydrated. Then, slides were incubated with 10% goat serum seal (Novus Biologicals, USA) solution at room temperature for 30 min. Next, slides were incubated with primary antibodies against p53 (1 : 500, Santa Cruz Biotechnology) and p21 (1 : 500, Santa Cruz Biotechnology) overnight at 4°C. Then, the slides were incubated with the second antibody (1 : 1000, Abcam, UK) at room temperature for 1 h. Enzyme conjugate was applied for 10 min at room temperature followed by development with AEC (Solarbio, Beijing, China). Each section was captured three times using a light microscope. For the negative control, PBS was used in place of primary antibody.

For IF assay, the 4 *μ*m sections were rehydrated. After rehydrated, 0.25% Triton X-100 in PBS was used as a membrane permeability agent. Then, sections were blocked with 10% goat serum seal solution at room temperature for 30 min. Next, slides were incubated with primary antibodies against Ki67 (1 : 1000, Thermo scientific) and Pax7 (1 : 250, Abcam, UK) overnight at 4°C, and then slides were incubated with anti-rabbit secondary antibody (1 : 10000, Abcam, UK) in the dark at room temperature for 1 h. Finally, sections were incubated with 4′,6-diamidino-2-phenylindole (1 : 10000, DAPI, Abcam, UK) for 10 min and photoed using fluorescence microscopy.

### 2.7. Immunofluorescence Assay of Cells

Cells were seeded in 24 well plates and stained in ice-cold 4% PFA at room temperature for 10 min on the third day. Cells were washed three times with PBS, and 0.25% Triton X-100 was used as membrane permeability agent. Next, cells were blocked with 5% bovine serum albumin (BSA) for 1 h and with primary antibodies against Ki67, Pax7, and HIF-1*α* (1 : 300, Novus Biologicals, USA) at 4°C for 48 h. Phosphate-buffered saline (PBS) is the control to primary antibody. Then, cells were washed three times with phosphate-buffered saline Tween-20 (PBST) and were incubated with a second antibody for 1 h at room temperature in the dark. At last, cells were incubated with DAPI for 8 min, and pictures were captured by fluorescence microscopy.

### 2.8. Quantitative Real-Time Polymerase Chain Reaction Assay

Total RNA was extracted from cells or tissues using TRIzol (Ambion, USA) reagent and then reverse transcribed to cDNA using PrimeScript RT reagent kit (Tiangen, China). Quantitative RT-PCR was performed with 20 *μ*l of reaction mixture containing SYBR Green PCR Master Mix (Light cycler, USA). Primer sequences of target genes are listed in Table 1. Relative expression level of each gene was calculated using the 2 − ΔΔCt methods. RNA expression was normalized to *β*-actin expression.

### 2.9. Western Blot Analysis

Cells were collected by 2x lysis buffer. Then, 30 *μ*l of total protein was separated on a 10% SDS-PAGE gel, and protein in the gel was transferred to a 0.45 *μ*m polyvinylidene difluoride (PVDF) membrane. Membrane was blocked by immersion in 5% milk for 1 h at room temperature. Next, membrane was incubated with primary antibodies against p53 (1 : 1000, Proteintech), p21 (1 : 1000, Abcam), MyHC (1:500, DSHB), MyoD (1 : 500, Millipore), and *β*-actin (1 : 10000, Absin, China) overnight at 4°C. After 4 × 6 min washes in TBST, secondary antibody (1 : 10000, Cell Signaling) was at room temperature for 2 h and the intensities of dies against p53 and p21 as a control for all other bands. Data were analyzed by Image J 1.5 software.

### 2.10. Statistical Analysis

The statistical analysis was performed by GraphPad Prism 7.0 (GraphPad Software, La Jolla, CA). All results were shown as mean ± SD from at least 3 independent experiments. *p* value was measured for the statistical significance of a two-tailed Student's *t*-test, and data were calculated by Excel. *p* < 0.05 was considered statistically significant.

## 3. Results and Discussion

### 3.1. Results

#### 3.1.1. The Structure and Function of Upper Airway Muscle Were Affected by Increasing Age

**Figure 1:**
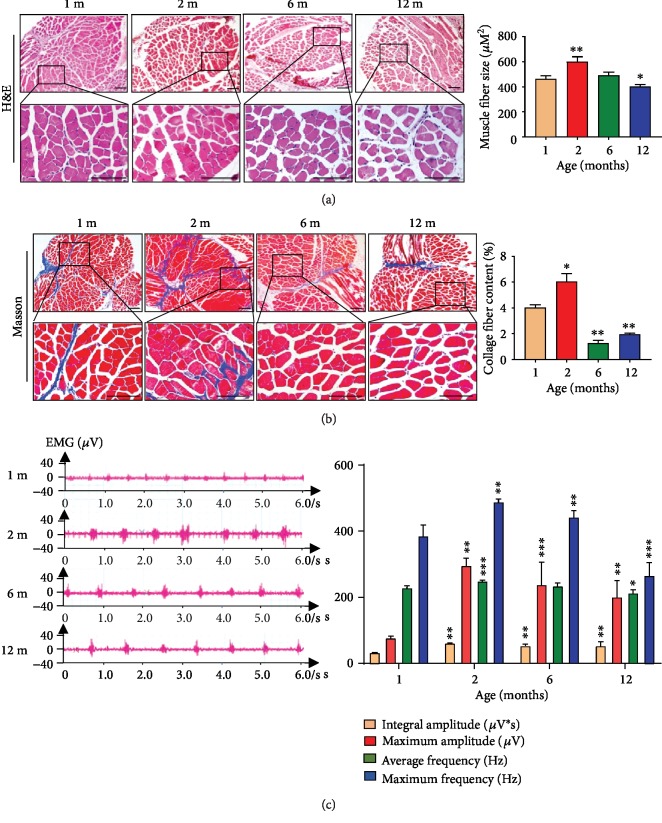
The structure and function of GG declined with aging. (a) The cross-sectional area of muscle fiber in 2-month-old significantly increased compared to other age groups. (b) The collagen content of 2-month-old was the highest among four age groups mice. (c) The genioglossus electromyographic activity in four age groups. ^∗^*p* < 0.05, ^∗∗^*p* < 0.01, and ^∗∗∗^*p* < 0.001. Scale bars are 100 *μ*m.

The upper airway becomes more collapsible with aging, and the genioglossus (GG) is the major upper airway muscle to maintain pharyngeal patency [6]. Therefore, the structure and function of GG play an important role in OSA. New generation fibers are related with muscle force deficit and fatigability [26]. Compared with other skeletal muscle, genioglossus has high specific gravity of the oxidized muscle fiber and is sensitive to oxygen [5]. To investigate whether GG muscle was altered by increasing age, we first examined the cross-sectional area (CSA) of muscle fibers which derived from four age groups. Our results showed that 6-month-old or 12-month-old mice had a CSA reduction compared to 2-month-old mice and a significantly less in 1-month-old compared to 2-month-old mice (Figure 1(a)). Similar results were found in collagen content of GG, which showed that 2-month-old mouse genioglossus has highest collagen content and less collagen content in 6 and 12-month-old mice compared to 2-month-old mice (Figure 1(b)).

Then, we used electromyography to analyze if muscle damage changes with increasing age. Electromyography is a kind of potential change that occurs when skeletal muscle is excited due to the generation, conduction, and diffusion of action potential of muscle fiber. We anesthetized mice of different ages (1-month-old, 2-month-old, 6-month-old, and 12-month-old) and then examined the genioglossus electromyographic activity (EMG_GG_) of these mice. The results showed that four indexes of EMG_GG_, including integral amplitude, maximum amplitude, average frequency, and maximum frequency, decreased with aging, except that 1-month-old was weakest (Figure 1(c)). These findings indicated that the structure and function of GG had significant reduction with increasing age in adult mice.

#### 3.1.2. The Renewal Ability Declined and p53/p21 Increased in Aging GG

**Figure 2:**
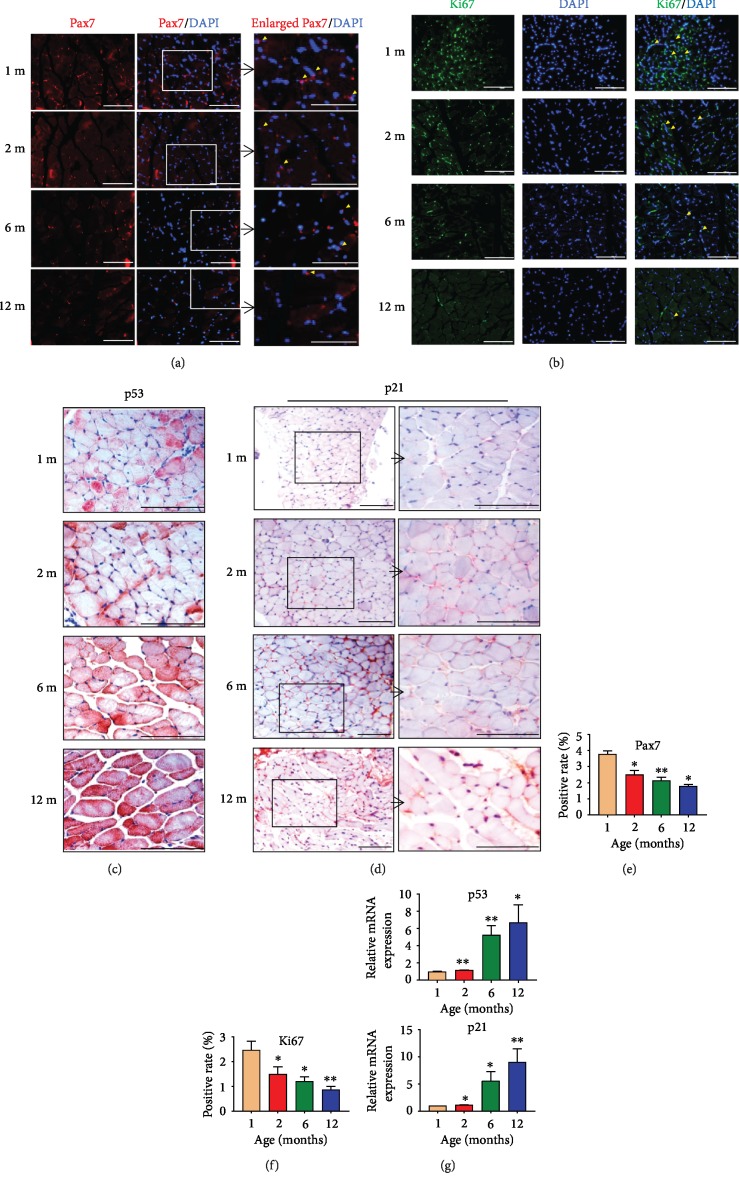
The renewal ability declined and senescence phenotype increased in aging GG. (a) Pax7, Pax7/DAPI, and enlarged Pax7/DAPI detected in GG muscles. (b) DAPI, Ki67, and Ki67/DAPI were detected in GG muscles. (c) The protein level of p53 was significantly increased in 12-month-old GG. (d) The protein level of p21 dramatically increased in 12-month-old GG compared to 1-month-old, 2-month-old, or 6-month-old. (e) Pax7-positive cells in GG significantly reduced in 12-month-old compared to 1-month-old. (f) Ki67-positive cells in GG greatly reduced in 12-month-old compared to 1-month-old. Ki67 was observed by immunofluorescence staining. (g) The mRNA levels of p53 and p21 also upregulated in aging GG. ^∗^*p* < 0.05 and ^∗∗^*p* < 0.01. Scale bars are 100 *μ*m.

In order to investigate whether increasing age affected the self-renewal function of MuSCs in GG, Pax7, the expression of the paired type homeobox transcription factor, was identified as a quantifiable marker for stem cells [27]. Therefore, we used Pax7 to detect the self-renewal function of GG MuSCs. Immunofluorescence assay showed that the Pax7-positive cells decreased gradually with aging (Figure 2(a)). We observed that the percentage of Pax7-positive cells in the GG were markedly reduced in 12-month-old (1.8%) compared to other age groups in Figure 2(e) (1 m: 3.7%; 2 m: 2.5%; and 6 m: 2.0%).

Similar results were found in proliferation ability of cells in GG. To investigate whether increasing age repaired the proliferation ability of cells in GG. Ki67, a marker protein for cell proliferation, was tested (Figure 2(b)). The results showed that Ki67-positive cells in the GG were reduced in 12-month-old (0.8%) compared with other age groups in Figure 2(f) (1 m: 2.5%; 2 m: 1.5%; and 6 m: 1.0%). Therefore, we concluded that the renewal function of GG decreased with aging.

Moreover, p53 and p21 are not only famous tumor suppressors but also members downstream of the aging and hypoxia signaling pathway [16, 17]. The senescence in GG was evaluated by the levels of p53 and p21. Immunohistochemistry analysis revealed that the protein levels of p53 and p21 were much higher in the GG of 12-month-old mice than young mice (Figures 2(c) and 2(d)). Meanwhile, p53 and p21 mRNA in 12-month-old were highest (p53 ≥ sixfold and p21 ≥ eightfold) among all age groups (Figure 2(g)). These findings discovered that p53 and p21 increased in aging GG.

#### 3.1.3. GG MuSCs Exhibited Worse Migration and Proliferation Abilities in Older Age

**Figure 3:**
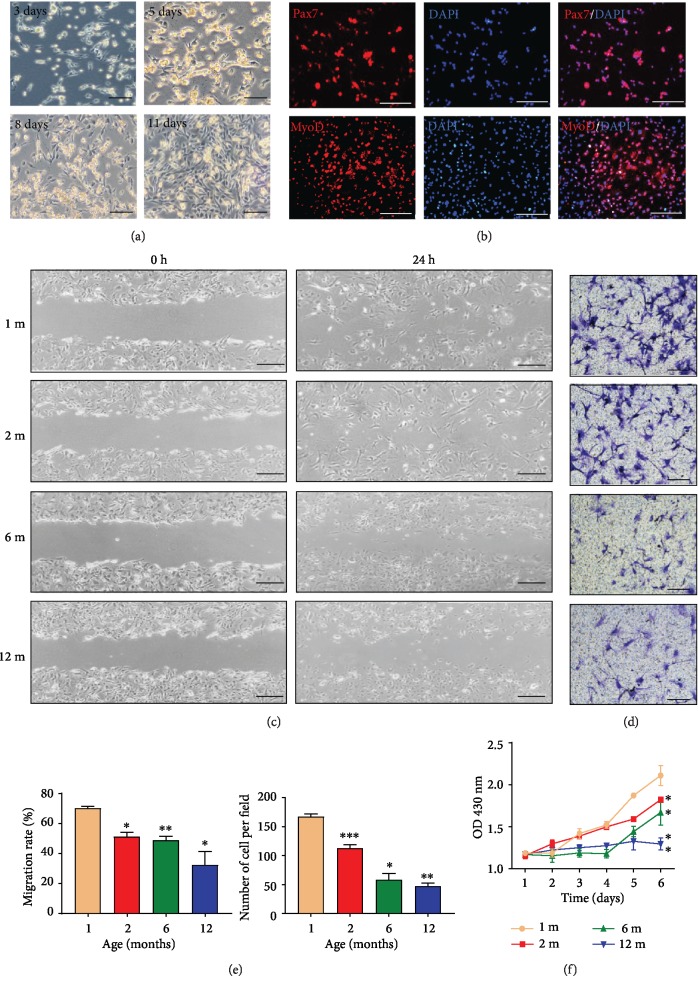
The proliferation and migration of GG MuSCs declined with aging. (a) Primary cells were obtained from GG muscle fibers, and cells showed a spindle morphology. (b) GG MuSC markers Pax7 and MyoD were positive in cells. (c) Wound healing of GG MuSCs derived from four age groups. (d) The migration of GG MuSCs derived from four age groups. (e) The wound healing and migration rate are significantly lower in 12-month-old compared to other age groups at 24 h. (f) The growth curve of GG MuSCs in four age groups. ^∗^*p* < 0.05 and ^∗∗^*p* < 0.01. Scale bars are 100 *μ*m.

OSA caused hypoxia and GG muscle injury. When the muscle is injured, MuSCs undergo differentiation into myocytes and fuse with each other in order to repair the injured muscle. Therefore, MuSCs are important functional cells in GG. To explore whether aging impaired GG MuSCs function, we derived GG MuSCs from four age groups of mice (Figure 3(a)). The cells were fixed after cultured 3 days. Immunofluorescence staining showed about 87% of isolated cells were Pax7-positive and 80% of cells were MyoD-positive (Figure 3(b)), which are the markers of MuSCs. The wound healing and the transwell migration chamber of GG MuSCs are shown (Figures 3(c) and 3(d)). Wound healing assay showed that the migration of GG MuSCs from 12-month-old was significantly slower than the other three groups (Figure 3(e)). The transwell migration chamber assay showed that GG MuSCs were all statistically significant among four age groups (Figure 3(e)). On the fourth day, the cell proliferation of 1-month-old mice was the fastest among the four age groups (Figure 3(f)). The GG MuSCs from 12 month-old mice exhibited decreased cell proliferation ability. These results suggested that aging impaired the migration and proliferation ability of GG MuSCs.

#### 3.1.4. p53/p21 Involved in Hypoxia and Aging Enhanced Hypoxia Response in GG MuSCs

**Figure 4:**
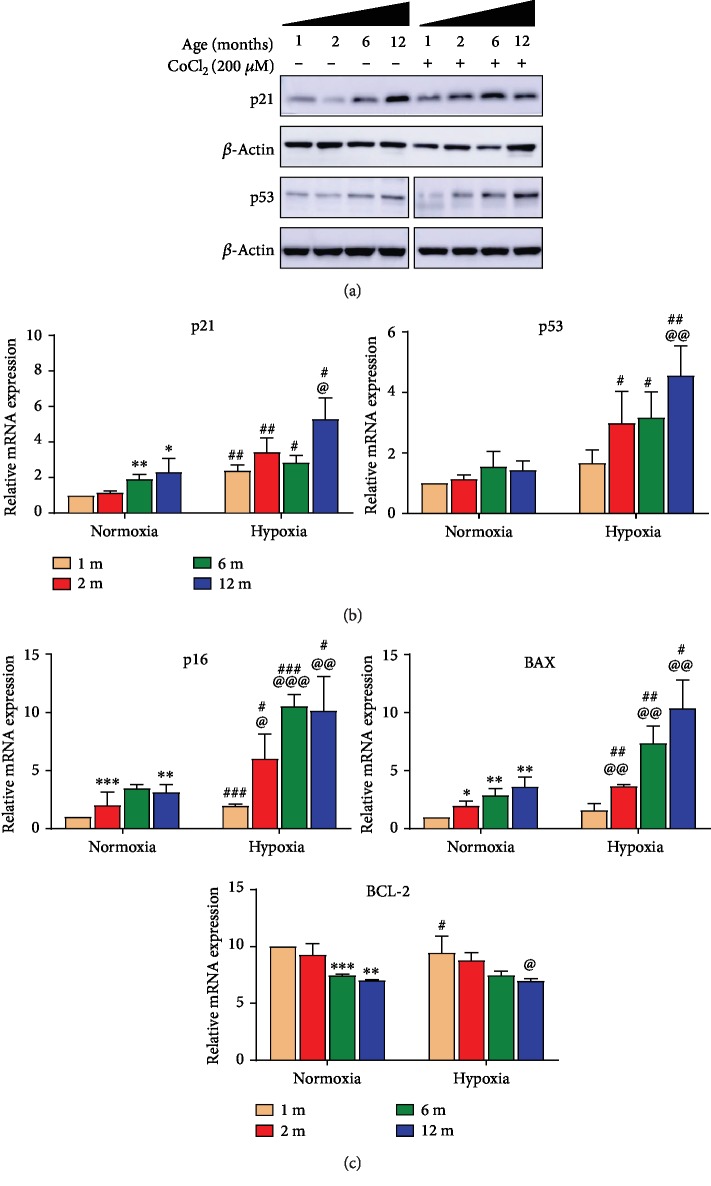
p53/p21 involved in hypoxia response in GG MuSCs. (a) The protein levels of p53 and p21 significantly increased under hypoxia, particularly in MuSCs derived from older age GG. (b) The mRNA levels of p53 and p21 also obviously grew up under hypoxia. The levels were higher in old GG MuSCs than in young. (c) The mRNA levels of p16 and BAX increased, while BCL-2 increased under hypoxia, especially in aging GG MuSCs. Statistical significance is marked as follows: ∗ represents the difference between normoxia and 1-month-old vs. 2-month-old vs. 6-month-old vs. 12-month-old; ^∗^*p* < 0.05, ^∗∗^*p* < 0.01, and ^∗∗∗^*p* < 0.001. @ represents the difference between hypoxia and 1-month-old vs. 2-month-old vs. 6-month-old vs. 12-month-old; ^@^*p* < 0.05, ^@@^*p* < 0.01, and ^@@@^*p* < 0.001. # represents difference between same age and normoxia vs. hypoxia; ^#^*p* < 0.05, ^##^*p* < 0.01, and ^###^*p* < 0.001.

OSA is characterized by hypoxia during sleep. In order to imitate the hypoxia of OSA, cells were treated with 0 *μ*M and 200 *μ*M CoCl_2_, a typical chemical hypoxia model. p53 and p21 are not only famous tumor suppressors but also belong to a downstream signaling pathway of aging and hypoxia [16, 17]. Firstly, we detected the levels of p53 and p21. The results showed that p53 and p21 increased in GG MuSCs under hypoxia. The levels of p53 and p21 severely increased in 12-month-old compared to 1-month-old (Figures 4(a) and 4(b)). Meanwhile, p16 and BAX increased in hypoxia, especially in aging GG MuSCs, while BCL-2 decreased (Figure 4(c)). These results suggested that p53, p21, and p16 involved the function regulation in GG MuSCs under hypoxia. These gene expressions were more obvious in GG MuSCs derived from aging muscle.

#### 3.1.5. Hypoxia Enhanced the Inhibitory Effect of Aging on GG MuSC Proliferation

**Figure 5:**
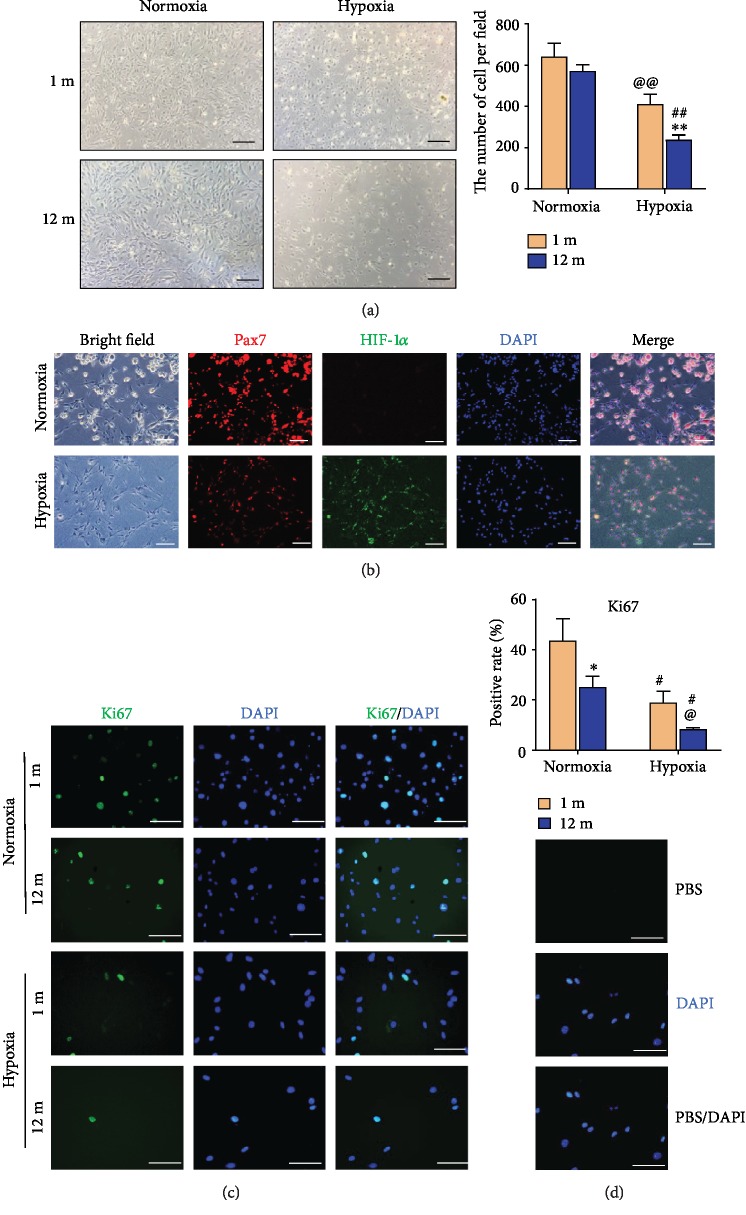
Hypoxia enhanced the inhibitory effect of aging on GG MuSC proliferation. (a) The GG MuSCs from 1-month-old and 12-month-old were treated with 0 *μ*M and 200 *μ*M CoCl_2_ for 3 days. The cell number was decreased to approximately 40% that of untreated cells. (b) Immunofluorescence staining for Pax7 and HIF-1*α* in MuSCs treated with or without CoCl_2_ for 3 days. (c) Ki67-positive cells in GG MuSCs under normoxia and hypoxia. Ki67 was observed by immunofluorescence staining. (d) The control experiment of IF. Statistical significance is marked as follows: ∗ represents difference between same CoCl_2_ and 1-month-old vs. 12-month-old; ^∗^*p* < 0.05, ^∗∗^*p* < 0.01, and ^∗∗∗^*p* < 0.001. @ represents the difference in 1-month-old and 0 vs. 200 *μ*M CoCl_2_; ^@^*p* < 0.05 and ^@@^*p* < 0.01. # represents the difference in 12-month-old and 0 vs. 200 *μ*M CoCl_2_; ^#^*p* < 0.05, ^##^*p* < 0.01, and ^###^*p* < 0.001. Scale bars are 100 *μ*m.

To explore whether hypoxia affected the proliferation of GG MuSCs, GG MuSCs were cultured under normoxia (0 *μ*M CoCl_2_) and hypoxia (200 *μ*M CoCl_2_). The results showed that the cell number under hypoxia was decreased to approximately 40% that of cells cultured under normoxia (Figure 5(a)). Then, we performed immunofluorescence staining for HIF-1*α,* the master transcription factor in response to cell hypoxia [28]. Our results showed that CoCl_2_ treatment induced high expression of HIF-1*α*. The data confirmed its nuclear localization in the CoCl_2_-treated groups, while no fluorescence was detected in control cells (Figure 5(b)). Meanwhile, the results showed that the percentage of Pax7-positive cells under hypoxia (87.78%) were higher than cells under normoxia (83.53%). It demonstrated that hypoxia promoted MuSCs self-renewal function (Figure 5(b)). Ki67 is a marker protein of ribosomal RNA transcription, which is necessary for cellular proliferation [29]. The results showed that Ki67-positive cells from 12-month-old were significantly reduced compared to 1-month-old under normoxia, and the decrease was higher than that observed under hypoxia (Figure 5(c)). The results of negative control group without primary antibody were shown (Figure 5(d)). Together, our data suggested the toxicity of hypoxia and implied that hypoxia decreased the proliferation and promoted self-renewal function of GG MuSCs, especially in older age cells.

#### 3.1.6. Hypoxia Strengthened the Influence of Aging in the Differentiation of GG MuSCs and Increased p53 and p21 Expression

**Figure 6:**
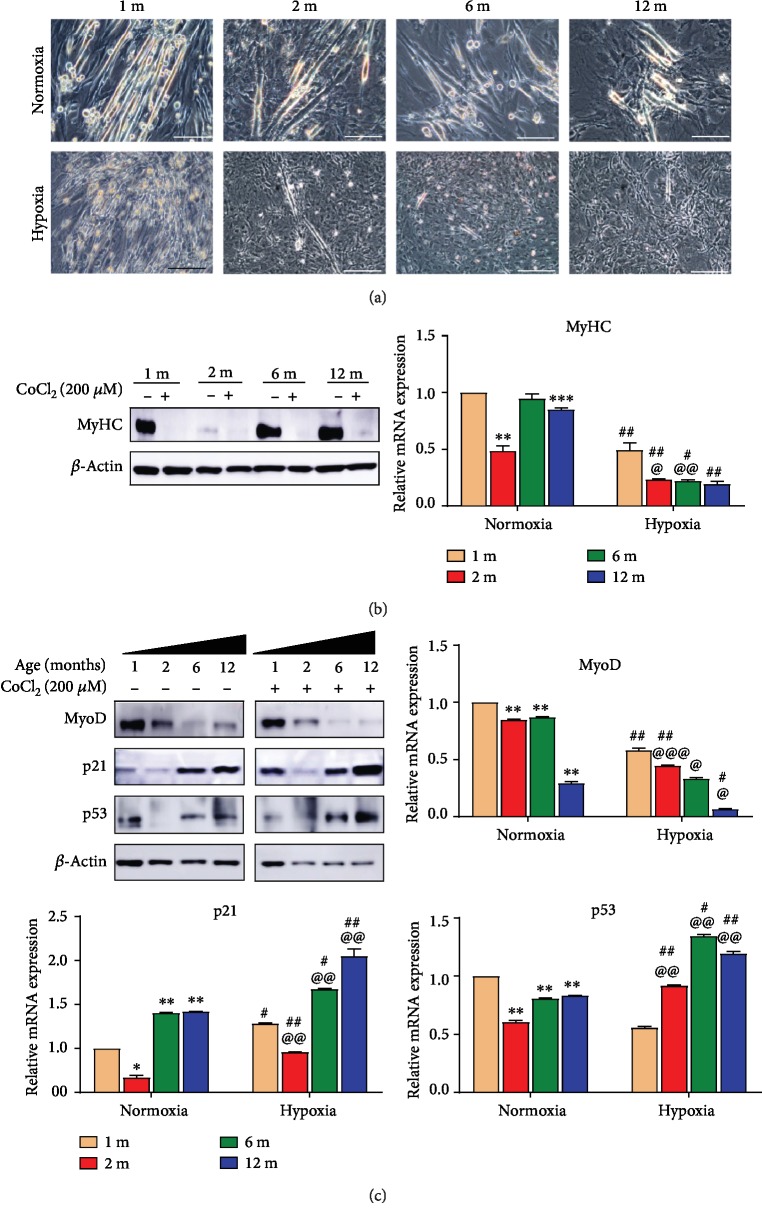
Hypoxia strengthened the influence of aging on GG MuSC differentiation. (a) The differentiation of GG MuSCs from four age groups under normoxia and hypoxia. (b) The expression level of MyHC was tested after differentiation in normoxia and hypoxia. (c) p53, p21, and MyoD were observed after differentiation under normoxia and hypoxia. p53 and p21 significantly increased, and the expressions of MyHC and MyoD were markedly downregulated under hypoxia. Aging further inhibited the levels of MyHC and MyoD and increased the levels of p53 and p21. Statistical significance is marked as follows: ∗ represents the difference between normoxia and 1-month-old vs. 2-month-old vs. 6-month-old vs. 12-month-old; ^∗^*p* < 0.05, ^∗∗^*p* < 0.01, and ^∗∗∗^*p* < 0.001. @ represents the difference between hypoxia and 1-month-old vs. 2-month-old vs. 6-month-old vs. 12-month-old; ^@^*p* < 0.05, ^@@^*p* < 0.01, and ^@@@^*p* < 0.001. # represents the difference between same age and normoxia vs. hypoxia; ^#^*p* < 0.05, ^##^*p* < 0.01, and ^###^*p* < 0.001. Scale bars are 100 *μ*m in all images. Scale bars are 100 *μ*m.

When GG muscles are damaged, GG MuSCs differentiate into myotubes, which plays an important role in repairing the damaged tissues [30]. To explore the relationship between aging and GG MuSCs differentiation, we examined the levels of MyHC and MyoD. The results showed that under hypoxia, the number of myotubes in GG MuSCs was much less than that under normoxia. Importantly, the number of myotubes was also less in aging GG MuSCs than in young (Figure 6(a)). With increasing aging, MyHC did not have significant change, but hypoxia inhibited the MyHC expression. MyoD is immensely suppressed by hypoxia, especially in aging cells (Figures 6(b) and 6(c)). Under hypoxia, p53 and p21 proteins were accumulated. This accumulation is more obvious in aging cells (Figure 6(c)). The mRNA p53 and p21 were significantly increased under hypoxia. MyHC and MyoD mRNA were also decreased, and the reduction was more severe in aging than young cells (Figure 6(c)). In conclusion, hypoxia aggravated the influence of aging on the proliferation and differentiation of GG MuSCs and p53 and p21 involved in the process.

## 4. Discussion

**Figure 7:**
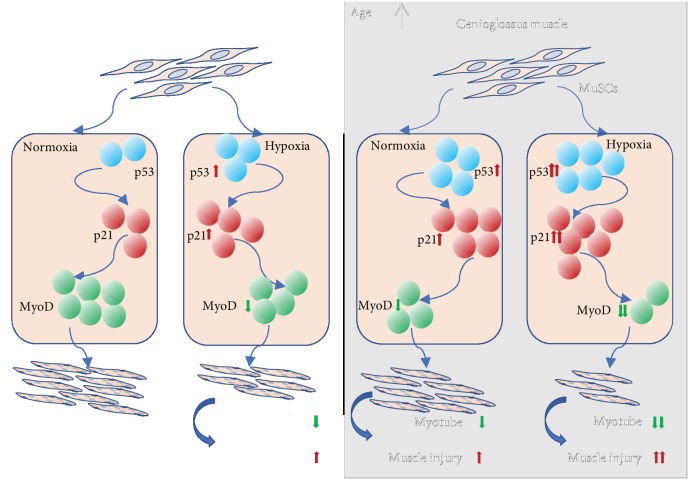
The diagrammatic sketch shows the damage mechanism of hypoxia and aging to genioglossus. Under hypoxia, increasing p53 and p21 induce the decline of MyoD. Then, low expression of MyoD leads to the reduction of myotube formation, especially in aging MuSCs. Finally, the results above enhance upper airway muscle injury.

The tissue homeostasis and organ function of multicellular organisms are gradually loss with aging. As aging, stem cell function has a progressive decline [31]. Therefore, the decrease of tissue homeostasis can be attributed to an age-related decline of stem cells [12]. The genioglossus, a major upper airway dilator, has abundant blood supply and high specific gravity of the oxidized muscle fiber and is sensitive to oxygen and rapid contraction compared with other skeletal muscles [5]. In this study, we characterized the effects of aging and hypoxia on GG injury and investigated p53/p21 role in this process (Figure 7).

We firstly investigated that the tissue function, such as cross-sectional area, collagen content, genioglossus electromyographic activity, the Pax7-positive cells and the Ki67 positive cells in genioglossus, which were decreased with aging. Both mRNA and protein p53 and p21 increased in GG muscle tissue. We also displayed that the proliferation and migration of GG MuSCs decreased with aging. Therefore, we suggest that aging impaired the GG and its MuSCs function. We further verified the influence of hypoxia in MuSCs derived from aging GG. Our data suggested that the migration, proliferation, and differentiation capacity of GG MuSCs declined with aging. Hypoxia further enhanced this inhibition effect by increasing the levels of p53 and p21.

Previous reports show that the upper airway becomes more collapsible with aging [5]. The type IIa fibers have a significant decrease, and IIb fibers have an increase in aging upper airway muscles [7]. There are some age-related changes and endurance in GG muscle fiber, and aged rats showed decreased susceptibility to hypoxia-induced stress [7, 32]. Our group has reported that hypoxia inhibits the GG MuSCs proliferation and differentiation [23, 33]. However, little attention is focused on the interacting of hypoxia and aging to vicious circle on upper airway muscles. Our study firstly demonstrated the mechanism of aging and hypoxia on upper airway in molecular level.

We demonstrated that the GG MuSCs from 2-month-old mice have some abnormal changes compared to other age groups. p53 and p21 were the lowest among four age groups. It may be related to adolescent behavior. During adolescence, the development of brain changes can be dynamic [34, 35]. Neurologically, synaptic and pruning myelination are associated with brain maturation. These processes are assumed to occur in accordance with macroscopic anatomical changes. GG muscle is innervated by the sublingual nerve. When hypoglossal nerve is stimulated, the physiologic state of GG muscle changes [36]. We speculated that the changes of GG MuSCs from 2-month-old mice might be due to the development of adolescent stimuli from the hypoglossal nerve.

Our results may rich the therapeutic theory and provide some treatment base for OSA patients in aging population. Clinically, patients with OSA are regularly treated with mechanical dilation of the upper airway to alleviate the symptoms of airway collapse. These mechanical dilation methods have many shortcomings such as poor patient compliance and more complications. The older have a poorer prognosis for the treatment of mechanical dilation of the upper airway especially. Therefore, more evidence is needed to elucidate the underlying mechanisms of interactions between aging and hypoxia on GG muscle.

Our results suggested that hypoxia and aging interact to form a vicious circle with upregulation of p53 and p21, and this vicious hypoxia plus aging damage accelerated upper airway muscle injury. However, we know little about the mechanism of neurological control of EMG_GG_. Altered neurological control of the GG may be the primary mechanism of OSA [37]. Therefore, age-related factors altering neural control of GG may be important in the elderly. Meanwhile, more studies are expected to explore the neurological control mechanism of the aging GG.

In summary, our study displayed that the function of GG muscles and MuSCs was affected by the aging process. Meanwhile, hypoxia aggravated the influence of aging in the proliferation and differentiation of GG MuSCs by increasing p53 and p21. Our findings highlight the important role of p53/p21 on the GG muscle during the aging process, and it may provide therapeutic basis in the repair of OSA upper airway injury.

## 5. Conclusions

OSA is a serious upper airway block problem. A population of more than 100 million is tolerant to this disease. OSA brings hypoxia and upper airway muscle injury. However, the damage mechanism of hypoxia and aging to the genioglossus is still unknown. In this study, we firstly discovered the effects of aging and hypoxia on GG MuSCs and showed detailed property analysis of mouse GG MuSCs. We found that aging affected the function of GG tissue and its MuSCs. Hypoxia suppressed the proliferation of mouse GG MuSCs, especially in MuSCs derived from aging GG, by increasing the expression levels of p53/p21. Identification of p53/p21 functions to mouse GG MuSCs may be helpful to understand cell senescence. Our study may benefit to reduce the airway obstruction and benefit the OSA therapies.

## Figures and Tables

**Table 1:** Primer sequences for qPCR.

Gene	Forward	Reverse
*β*-Actin	GTGACGTTGACATCCGTAAAGA	GCCGGACTCATCGTACTCC
p53	CCCCTGTCATCTTTTGTCCCT	AGCTGGCAGAATAGCTTATTGAG
p21	CGAGAACGGTGGAACTTTGAC	CCAGGGCTCAGGTAGACCTT
p16	GCTCAACTACGGTGCAGATTC	GCACGATGTCTTGATGTCCC
MyHC	GCGAATCGAGGCTCAGAACAA	GTAGTTCCGCCTTCGGTCTTG
MyoD	CGGGACATAGACTTGACAGGC	TCGAAACACGGGTCATCATAGA
BAX	AGACAGGGGCCTTTTTGCTAC	GTAGTTCCGCCTTCGGTCTTG
BCL-2	GCTACCGTCGTGACTTCGC	CCCCACCGAACTCAAAGAAGG

## Data Availability

The data used to support the findings of this study are included within the article.
